# Supplementary material for: Thermophoretic glycan profiling of extracellular vesicles for triple-negative breast cancer management
Source: Nat Commun. 2024 Mar 14;15:2292. doi: 10.1038/s41467-024-46557-5 (PMC10937950; doi:10.1038/s41467-024-46557-5)
Supplement: Supplementary file 1 — Supplementary Information [file 41467_2024_46557_MOESM1_ESM.pdf]

SUPPLEMENTARY INFORMATION

Thermophoretic Glycan Profiling of Extracellular Vesicles for Triple-Negative Breast Cancer Management

Li et al.

Supplementary Information Contains:

Supplementary Figures 1-34

Supplementary Tables 1-9

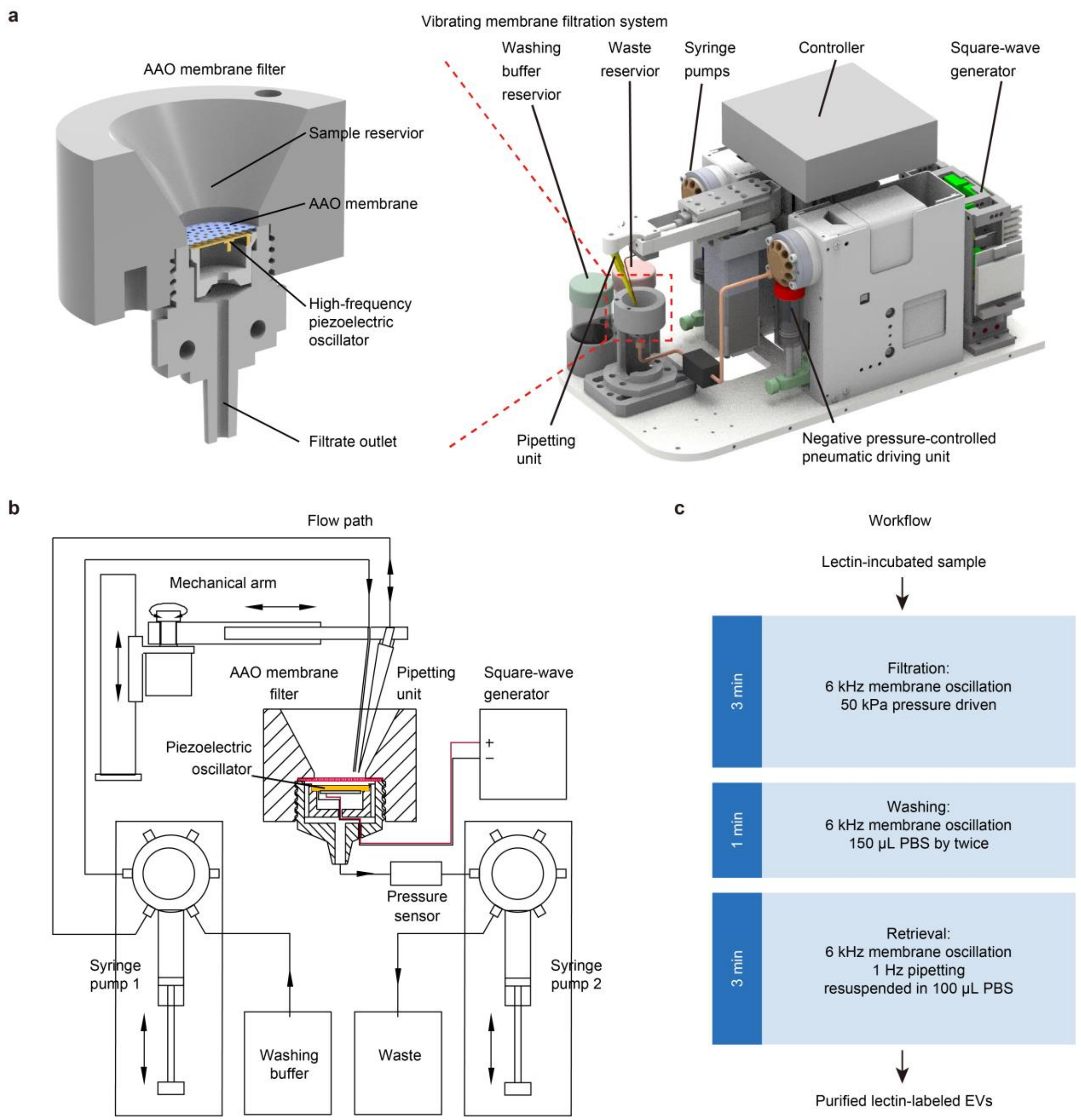

Supplementary Fig. 1 | Customized vibrating membrane filtration (VMF) system for automatic filtration, washing and retrieval of EVs. a, Design of the customized VMF system. The major components included an AAO membrane filter, a high-frequency piezoelectric oscillator, a negative pressure-controlled pneumatic driving unit and a pipetting unit. **b,** Flow path of the filtration system. **c,** Workflow of automatic filtration, washing and retrieval of EVs using the filtration system within 10 min.

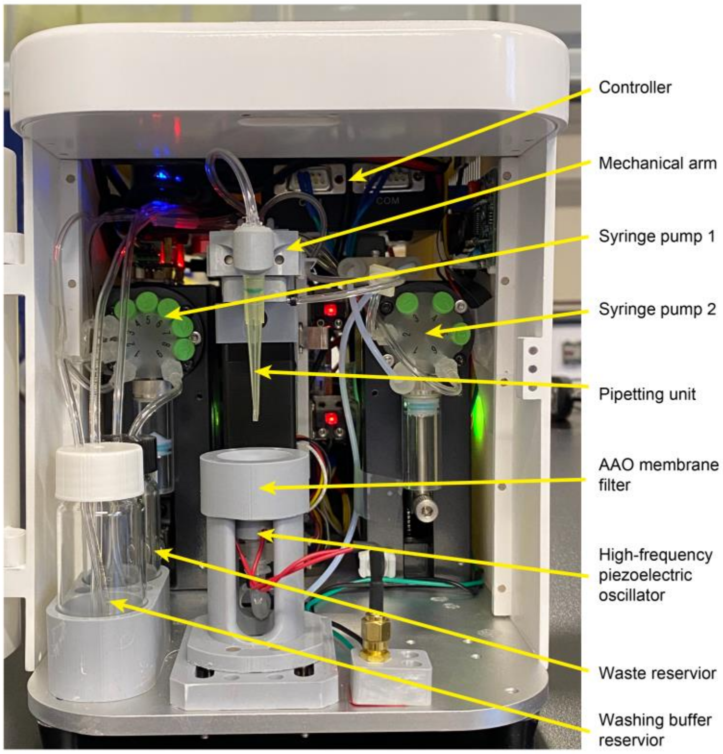

Supplementary Fig. 2 | Photograph of VMF system.

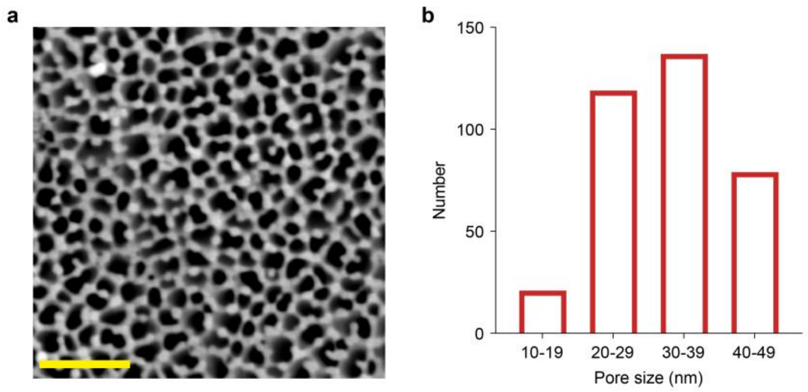

Supplementary Fig. 3 | Characterization of pore size of the AAO membrane. a, SEM image of AAO membrane with a nominal pore diameter of 20 nm. The representative images are shown from three independent repeats. Scale bar, 200 nm. **b,** Size distribution of nanopores in the AAO membrane (*n* = 385 pores) obtained by ImageJ. Source data are provided as a Source Data file.

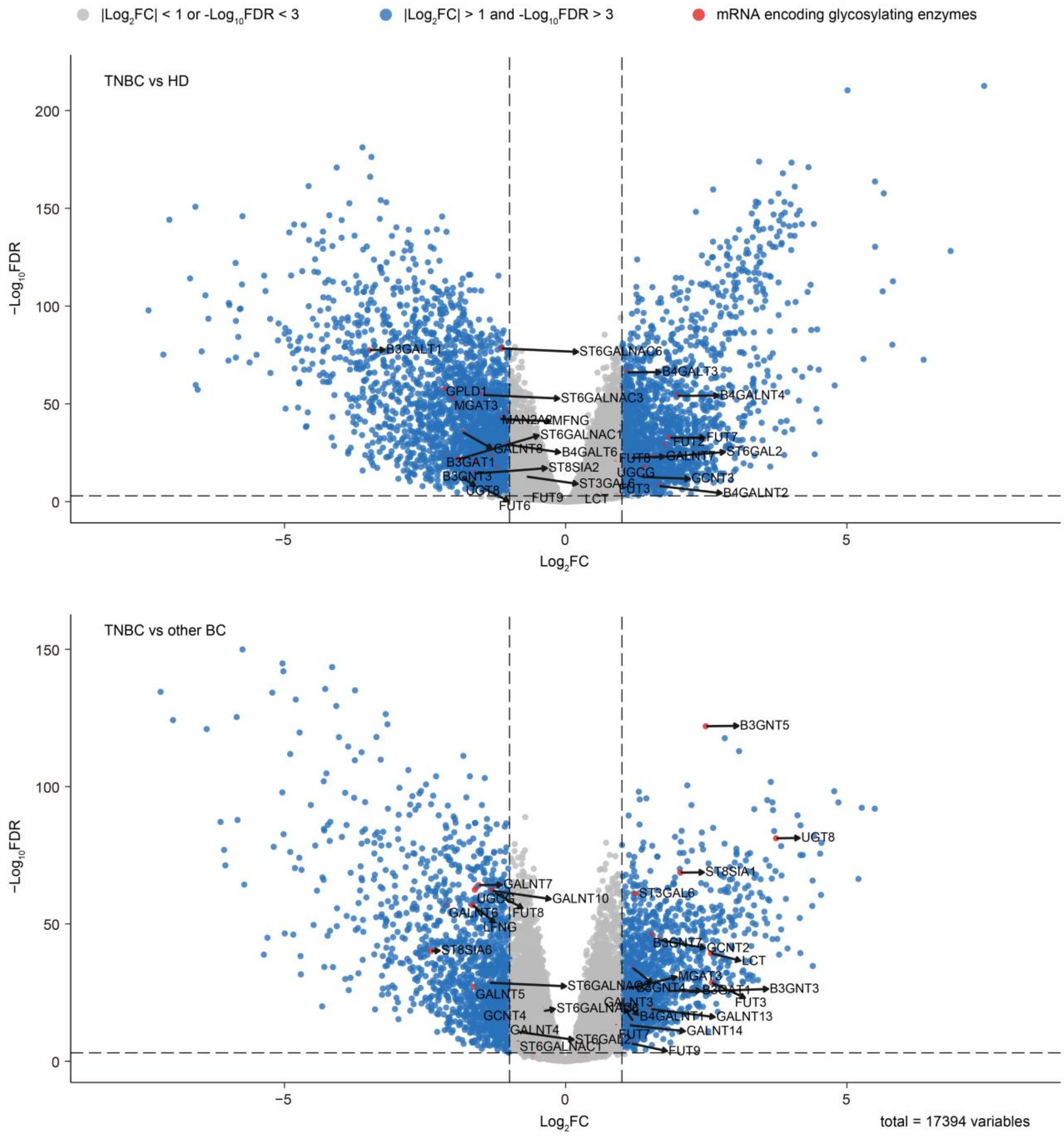

Supplementary Fig. 4 | Identification of TNBC-associated mRNAs by analysis of TCGA-BRCA database. TNBC-associated mRNAs were those differentially expressed either between TNBC and HD (top) or between TNBC and other BC subtypes (bottom) by differential expression analysis. The mRNAs encoding glycosyltransferases or glycosidases that involved in the biosynthesis of glycans are indicated by red dots. FC, fold change. FDR, false discovery rate. Source data are provided as a Source Data file.

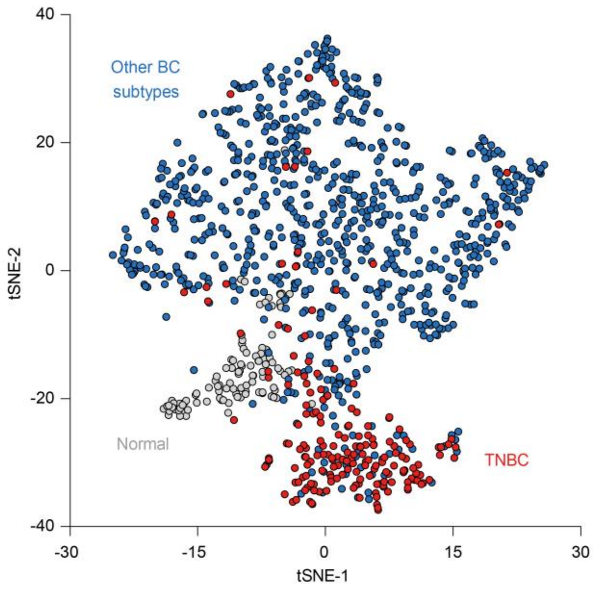

Supplementary Fig. 5 | t-SNE plot of mRNAs encoding glycosyltransferases or glycosidases showing the discrimination of TNBC, other BC subtypes and normal tissues. Gray dots represent normal tissue (*n* = 113), blue dots represent other BC subtype (*n* = 862), red dots represent TNBC (*n* = 178). Source data are provided as a Source Data file.

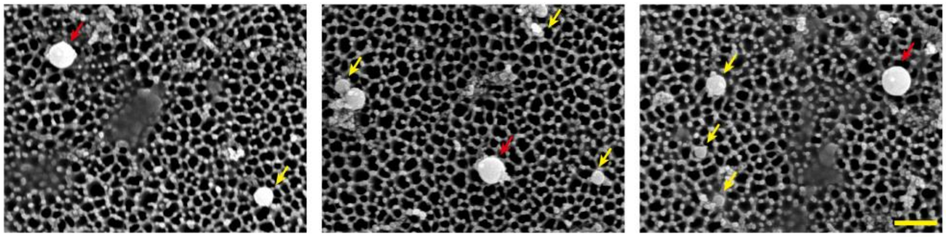

Supplementary Fig. 6 | SEM images of MDA-MB-231 EVs on AAO membranes after VMF. 200 μL of MDA-MB-231 EVs (10^7 μL^{-1}) was loaded onto the membrane filter. EVs smaller than 100 nm were indicated by yellow arrows, EVs larger than 100 nm were indicated by red arrows. Scale bar, 200 nm.

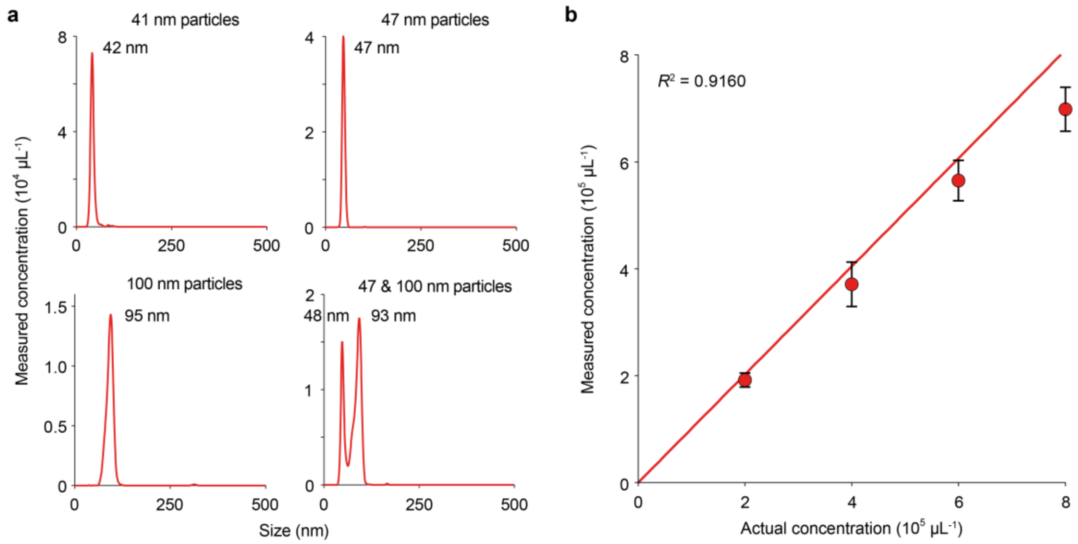

Supplementary Fig. 7 | Size detection limit of NTA. a, NTA measurement of size distribution of polystyrene (PS) particles of nominal diameters of 41 nm, 47 nm and 100 nm, and a mixture of 47 nm and 100 nm particles. Size modes are indicated. **b**, NTA measurement of 47 nm particles with varied concentrations (n = 3, mean ± s.d.). R square (R^2) is indicated. Error bars represent the mean ± s.d. in (**b**). Source data are provided as a Source Data file.

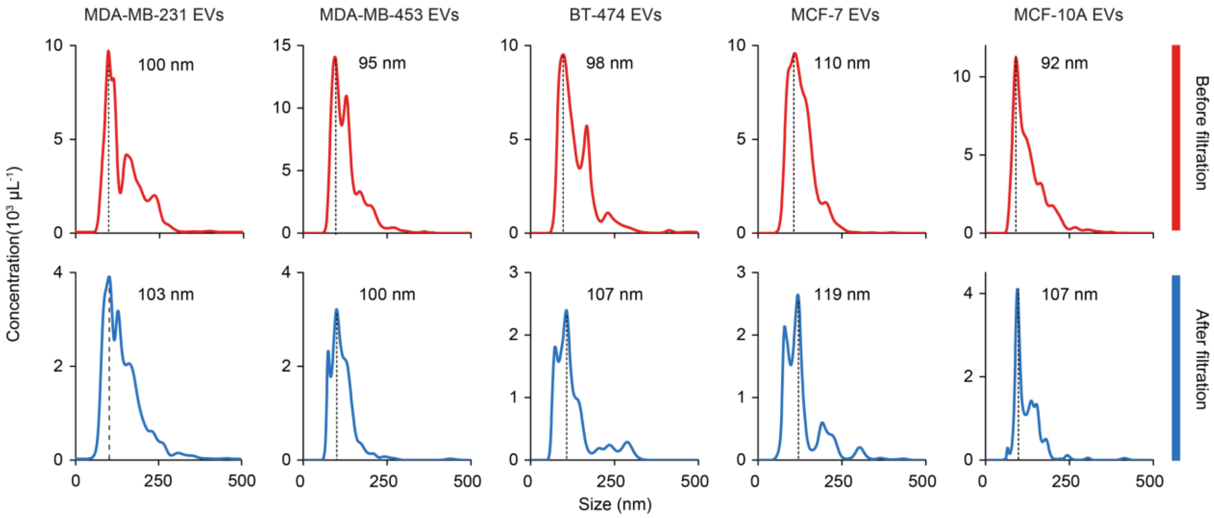

Supplementary Fig. 8 | NTA characterization of size distribution and concentration of EVs before and after VMF. The equal concentration of EV (10^7 μL^{-1}) was used across different EV types. Size modes are indicated. Source data are provided as a Source Data file.

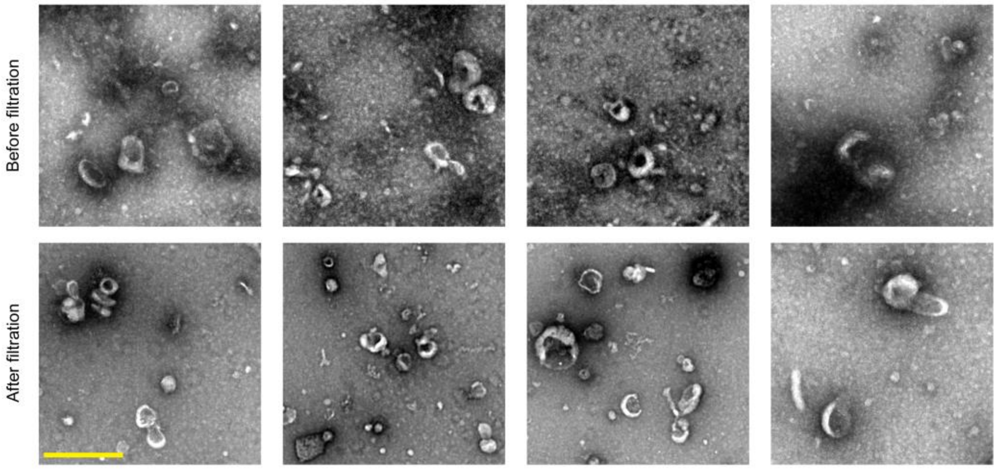

Supplementary Fig. 9 | TEM images of MDA-MB-231 EVs before and after VMF. 200 μL of MDA-MB-231 EVs (10^7 μL^{-1}) was loaded onto the membrane filter. Scale bar, 500 nm.

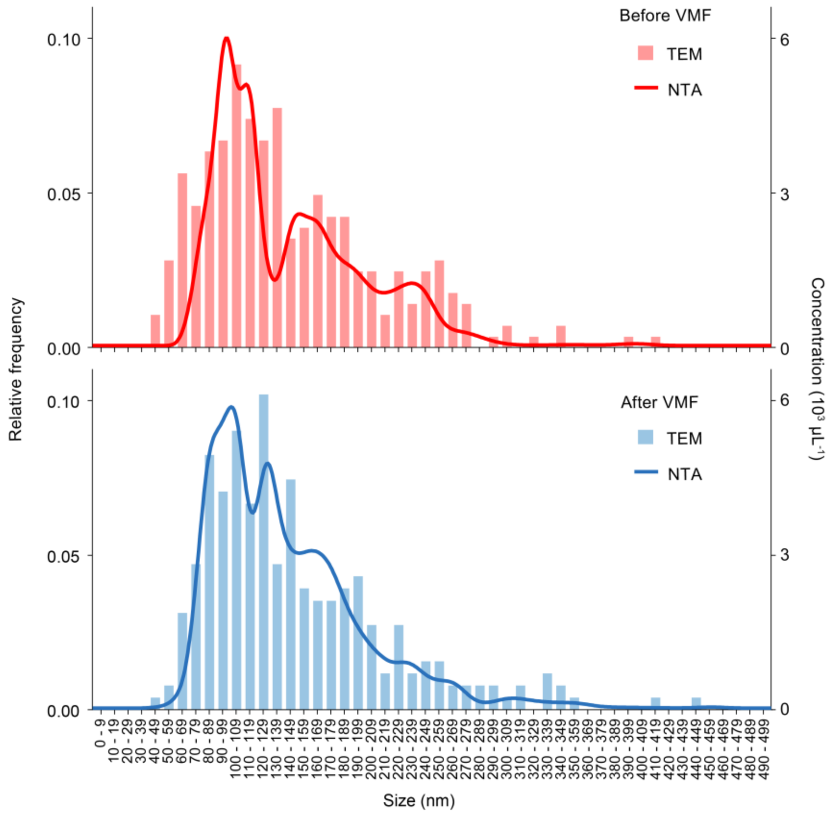

Supplementary Fig. 10 | Size distribution of MDA-MB-231 EVs. TEM (bar) and NTA (line) showing the size distribution of MDA-MB-231 EVs before and after VMF. TEM characterization indicating that 27 % of EVs before VMF and 24 % of EVs after VMF were smaller than 100 nm (n = 284 EVs before VMF, n = 255 EVs after VMF). Source data are provided as a Source Data file.

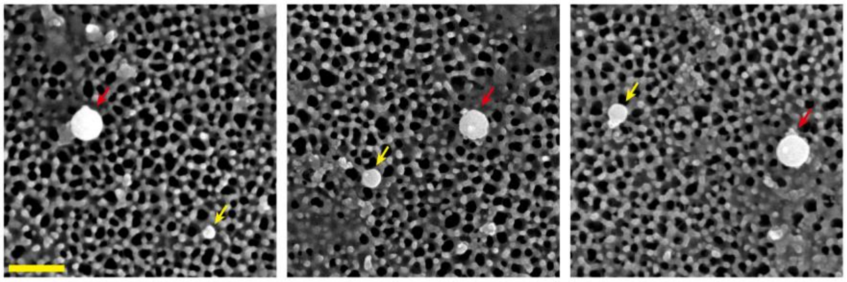

Supplementary Fig. 11 | SEM images of plasma EVs on AAO membranes after VMF. Human plasma (2 μL, diluted by 750 folds) was loaded onto the membrane filter. EVs smaller than 100 nm were indicated by yellow arrows, EVs larger than 100 nm were indicated by red arrows. Scale bar, 200 nm.

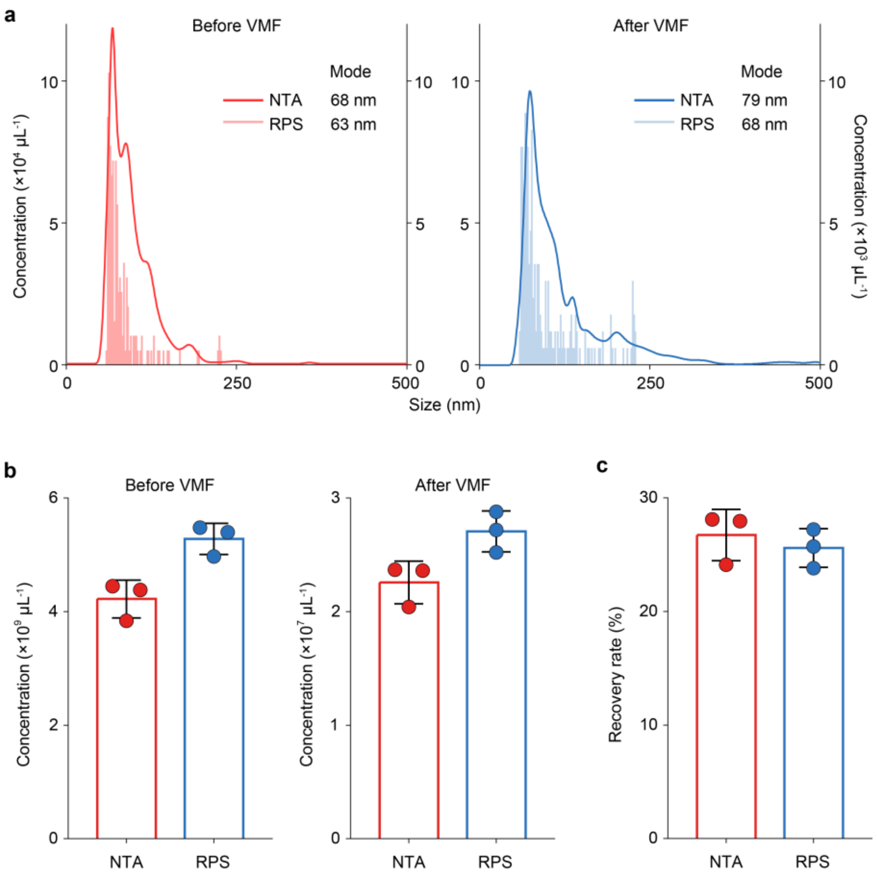

Supplementary Fig. 12 | Characterization of size distribution and concentration of bioparticles by RPS and NTA. a, Similar size distributions for a plasma sample before and after VMF as determined by NTA (lines) and RPS (bars). Size modes are indicated. **b,** Particle concentrations before and after VMF measured by NTA and RPS (the same sample was measured repeatedly for 3 times). **c,** Recovery rates calculated based on the concentrations in (**b**) (the same sample was measured repeatedly for 3 times). A resistive pulse sensor (Resun Technology, Co., Ltd, Shenzhen, China) was used to detect the size distribution and concentration of bioparticles. RPS was equipped with a custom chip whose measurement range was suitable for the analytes. Error bars represent the mean ± s.d. in (**b**, **c**). Source data are provided as a Source Data file.

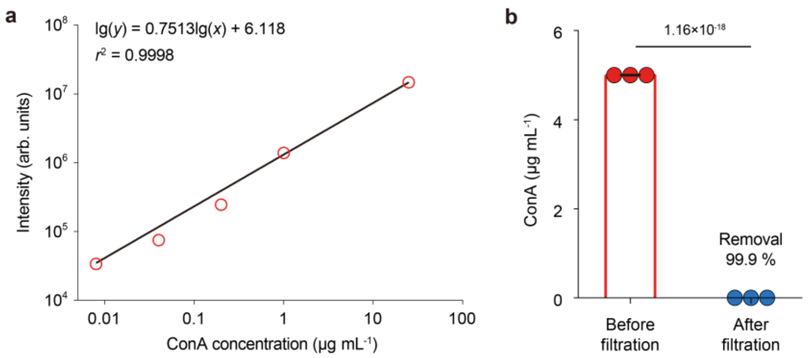

Supplementary Fig. 13 | Quantification of removal rate of free FITC-ConA (without the presence of EVs) by VMF. a, Standard curve for calculating the removal rate of free lectin after VMF. The linear range of detection of ConA by fluorescence spectrometer was 0.008 – 25 μg mL^{-1} (n = 3 samples for each ConA concentration). R square (r^2) is indicated. **b,** The concentration of free FITC-ConA before or after VMF. 5 μg mL^{-1} ConA solution was used as the input and the concentration of ConA in the filtrate was quantified based on the standard curve (n = 3 samples for each condition). Statistical difference was determined by a two-sided, parametric *t*-test (**b**). *P* value is indicated. Error bars represent the mean ± s.d. in (**a**, **b**). Source data are provided as a Source Data file.

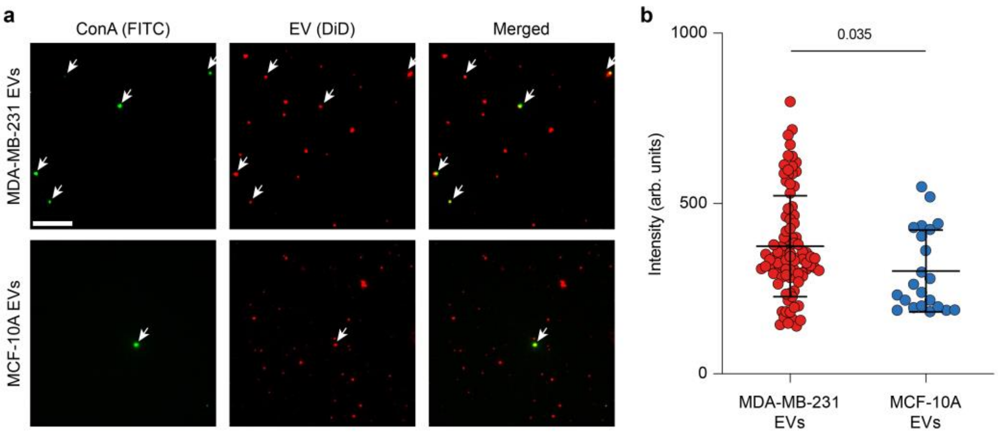

Supplementary Fig. 14 | Fluorescence colocalization of DiD- and Con A (FITC)-labeled EVs. **a**, MDA-MB-231 EVs and MCF-10A EVs subjected to ConA labeling, VMF and DiD labeling before fluorescence microscopy observation. The representative images are shown from three independent repeats. Scale bar, 20 µm. **b**, FITC fluorescence intensity of 90 DiD-FITC colocalization events for MDA-MB-231 EVs and 22 events for MCF-10A EVs. Statistical differences were determined by two-sided, nonparametric Mann–Whitney test (**b**). *P* value is indicated in the chart. Error bars represent the mean ± s.d. in (**b**). Source data are provided as a Source Data file.

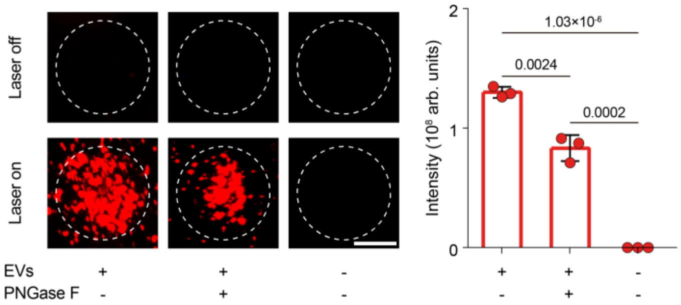

Supplementary Fig. 15 | Specificity of ConA for detecting EV glycans. Fluorescence images were taken for ConA-labeled MDA-MB-231 EVs, ConA-labeled MDA-MB-231 EVs after PNGase F treatment, and negative control sample (free ConA without EVs) before and after thermophoretic accumulation. The representative images are shown from three independent repeats. Scale bar, 50 µm. Quantification of fluorescence intensities of different samples after thermophoretic accumulation was shown in the right panel (n = 3 sample for each condition). Statistical difference was determined by a two-sided, parametric t-test. P values are indicated. Error bars represent the mean ± s.d. Source data are provided as a Source Data file.

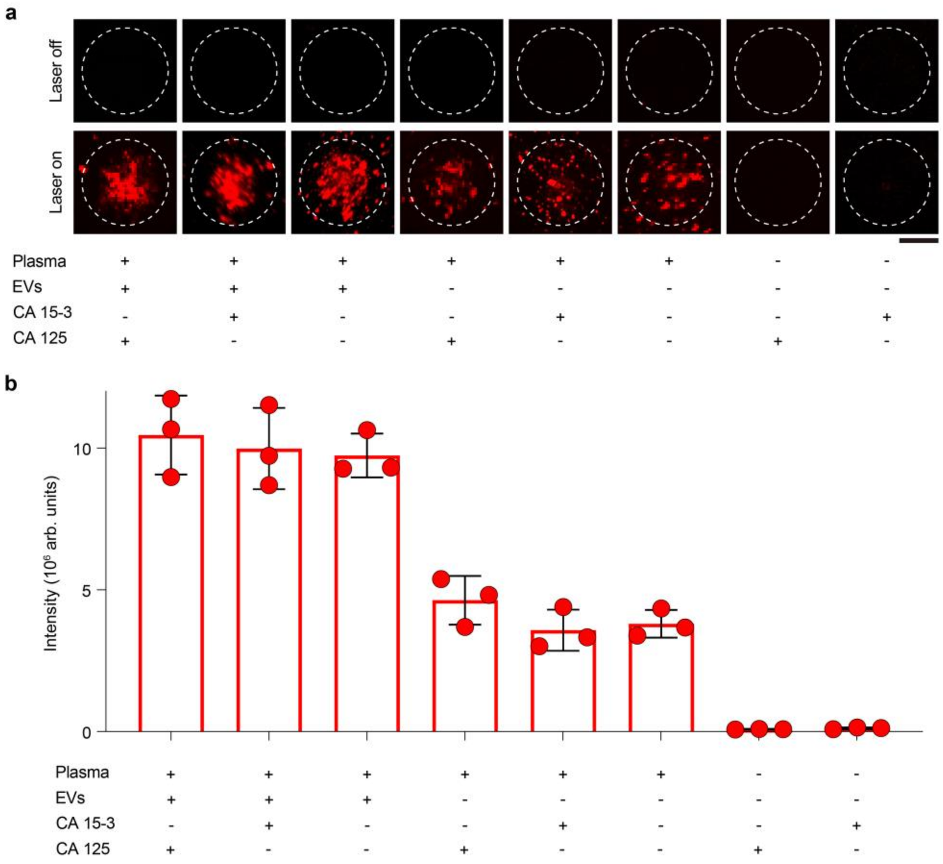

Supplementary Fig. 16 | Minimal effect of soluble protein CA 125 or CA15-3 on EV glycan detection. a, Fluorescence images of 8 types of samples measured by EVLET using FITC-ConA. (1) HD plasma spiked with CA 125 (35 U mL^{-1}) and MDA-MB-231 EVs (1.4×10^{8} EVs per assay); (2) HD plasma spiked with CA 15-3 (25 U mL^{-1}) and MDA-MB-231 EVs (1.4×10^{8} EVs per assay); (3) HD plasma spiked with MDA-MB-231 EVs (1.4×10^{8} EVs per assay); (4) HD plasma spiked with CA 125 (35 U mL^{-1}); (5) HD plasma spiked with CA 15-3 (25 U mL^{-1}); (6) HD plasma alone; (7) CA 125 (35 U mL^{-1}); (8) CA 15-3 (25 U mL^{-1}). The representative images are shown from three independent repeats. Scale bar, 50 µm. **b**, Quantification of fluorescence intensities of different samples after thermophoretic accumulation (n = 3 samples for each condition). Error bars represent the mean ± s.d. Source data are provided as a Source Data file.

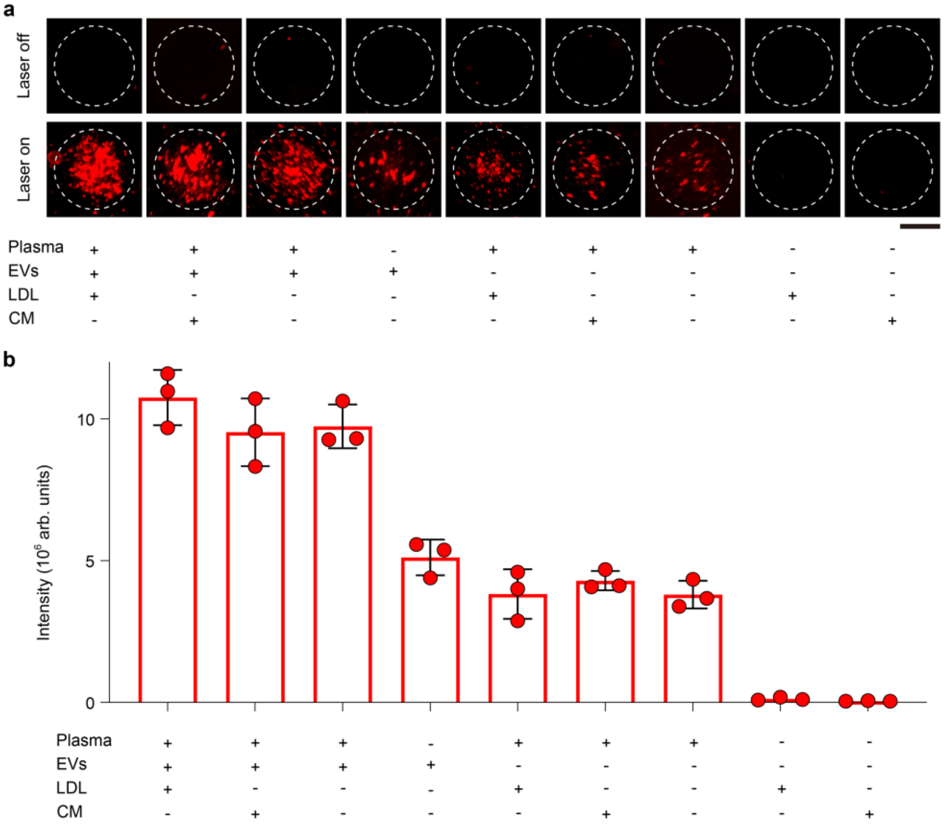

Supplementary Fig. 17 | Minimal effect of lipoproteins on EV glycan detection. a, Fluorescence intensities of 9 types of samples measured by EVLET: (1) HD plasma spiked with MDA-MB-231 EVs (1.4×10^{8} EVs per assay) and LDL (2.7 mg mL^{-1}); (2) HD plasma spiked with MDA-MB-231 EVs and CM (1.3 mg mL^{-1}); (3) HD plasma spiked with MDA-MB-231 EVs; (4) MDA-MB-231 EVs; (5) HD plasma spiked with LDL; (6) HD plasma spiked with CM; (7) HD plasma; (8) LDL; (9) CM (n = 3 samples for each condition). **b**, Quantification of fluorescence intensities of different samples after thermophoretic accumulation (n = 3 samples for each condition). Error bars represent the mean ± s.d. Source data are provided as a Source Data file.

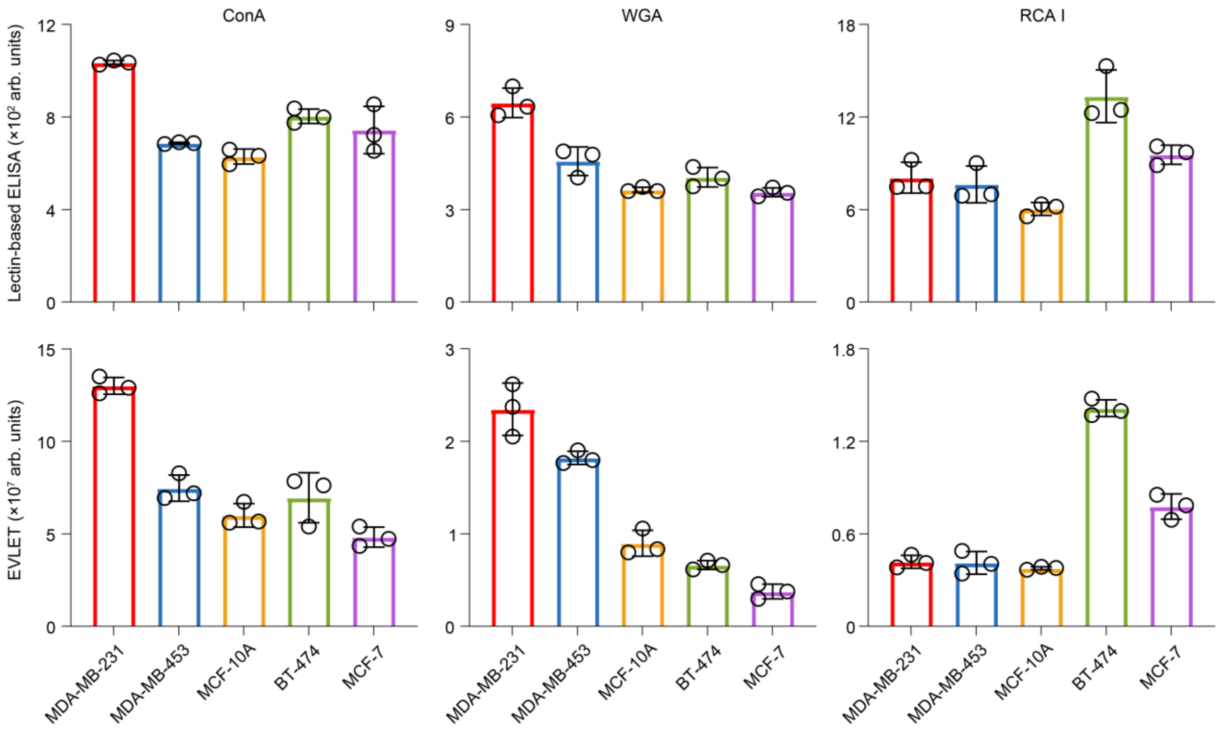

Supplementary Fig. 18 | Glycan patterns of cell line EVs. EV glycans measured by lectin-based ELISA (top row) and EVLET (bottom row) using ConA, WGA and RCA I (*n* = 3 samples for each cell line EVs). Error bars represent the mean ± s.d. Source data are provided as a Source Data file.

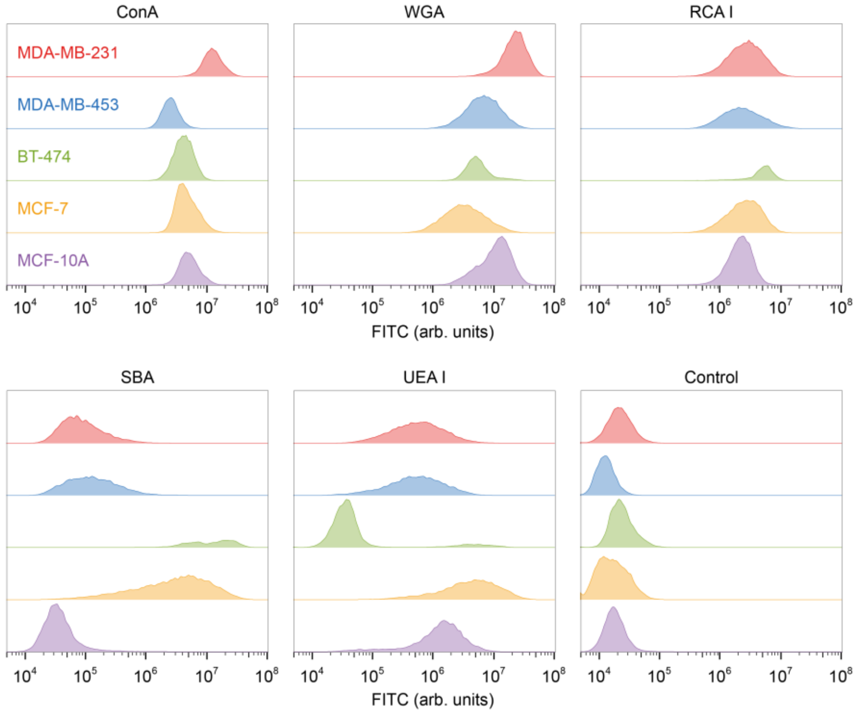

Supplementary Fig. 19 | Glycan patterns of breast cell lines. Flow cytometric analysis of surface glycans on 5 breast cell lines (MDA-MB-231, MDA-MB-453, BT-474, MCF-7 and MCF-10A) after labeling by a panel of FITC-conjugated lectins (ConA, WGA, RCA I, SBA and UEA I). Cells without lectin labeling were used as the control.

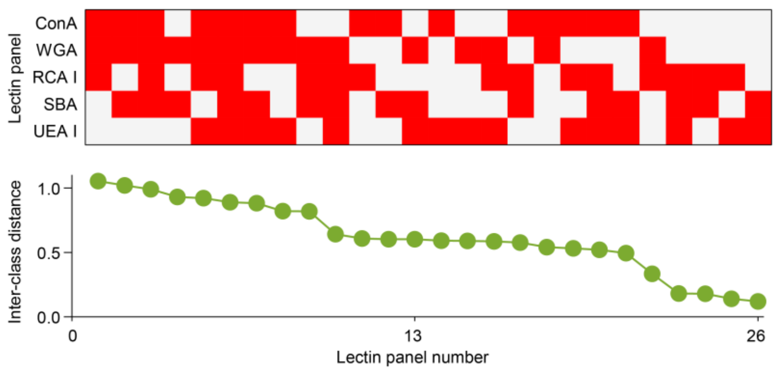

Supplementary Fig. 20 | The inter-class distance across the 3 groups (TNBC EVs, other BC EVs and benign EVs) calculated for all possible combinations of 3 – 5 lectins based on principle component analysis (PCA). Source data are provided as a Source Data file.

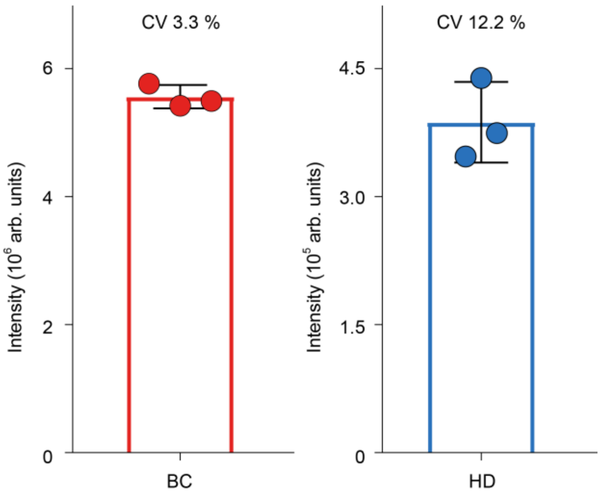

Supplementary Fig. 21 | Reproducibility of EVLET for detecting plasma EV glycans. WGA-labeled EVs were measured for one BC plasma sample and one HD plasma sample at different time points (the same sample was measured repeatedly for 3 times). CV (coefficient of variation) values are indicated. Error bars represent the mean ± s.d. Source data are provided as a Source Data file.

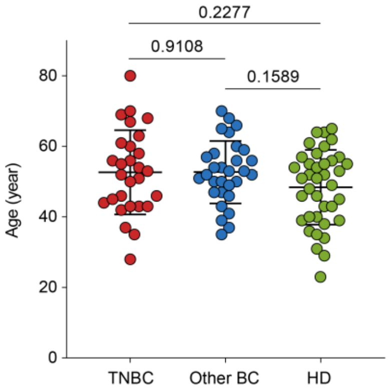

Supplementary Fig. 22 | Comparison of age distribution across TNBC (*n* = 28), other BC (*n* = 30) and HD (*n* = 38) cohorts. No statistically significant difference was observed between different cohorts. Statistical differences were determined by two-sided, nonparametric Mann–Whitney test. *P* values are indicated in the chart. Error bars represent the mean ± s.d. Source data are provided as a Source Data file.

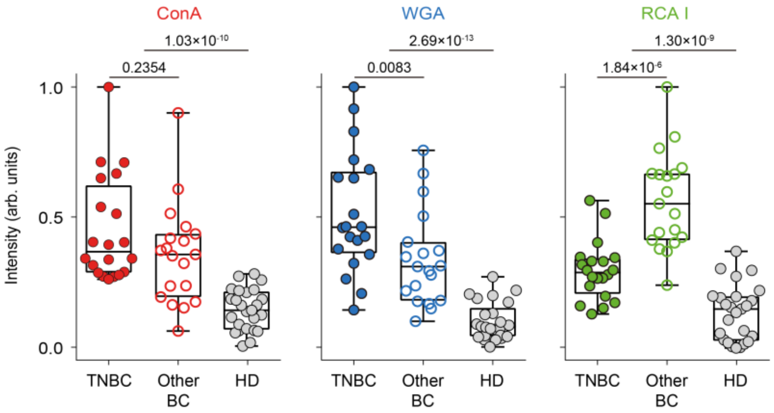

Supplementary Fig. 23 | Heterogeneous expression levels of lectin-labeled EVs in the training cohort. The expression levels of lectin-labeled EVs vary across TNBC (n = 20), other BC subtypes (n = 19) and HD (n = 25). Statistical differences were determined by two-sided, nonparametric Mann–Whitney test. P values are indicated in the chart. The central line, box and error bar indicate the median, inter-quartile range (Q1 and Q3) and min-max range, respectively. Source data are provided as a Source Data file.

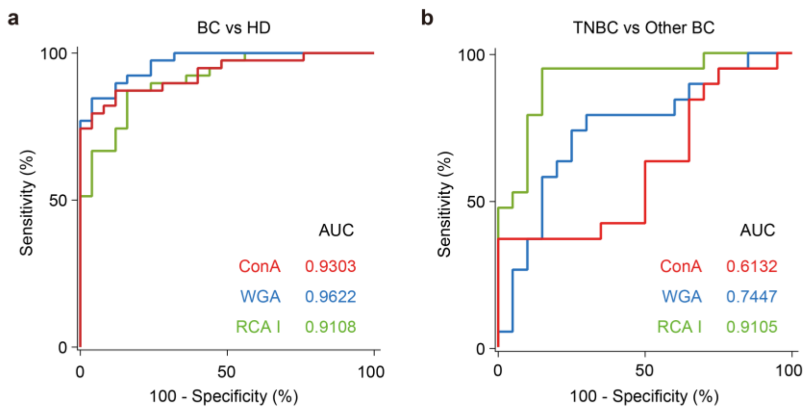

Supplementary Fig. 24 | ROC curves of individual lectins in TNBC diagnosis within the training cohort. a, ROC curves of ConA, WGA and RCA I for discrimination between BC and HD. **b,** ROC curves of ConA, WGA and RCA I for discrimination between TNBC and other BC subtypes. Source data are provided as a Source Data file.

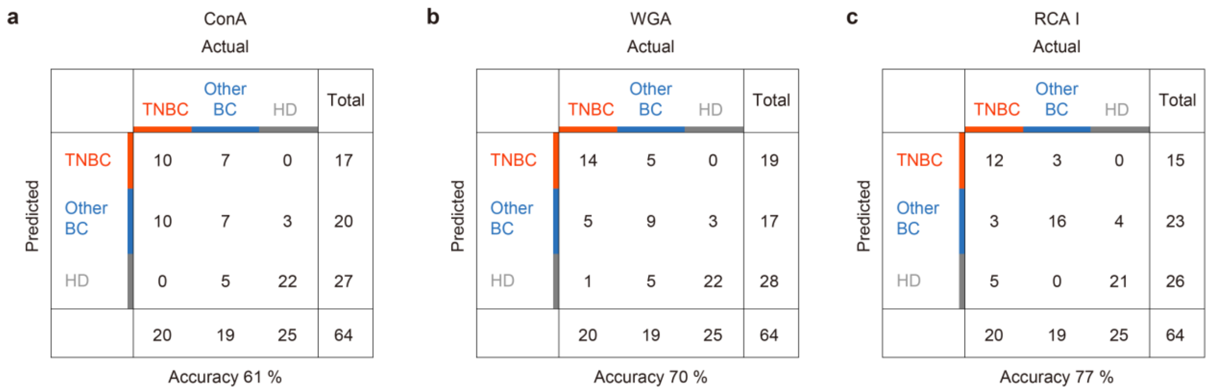

Supplementary Fig. 25 | Confusion matrix of individual lectins in discrimination across TNBC, other BC subtypes and HD within the training cohort. Confusion matrix of ConA (**a**), WGA (**b**) and RCA I (**c**) for classifying TNBC, other BC subtypes and HD.

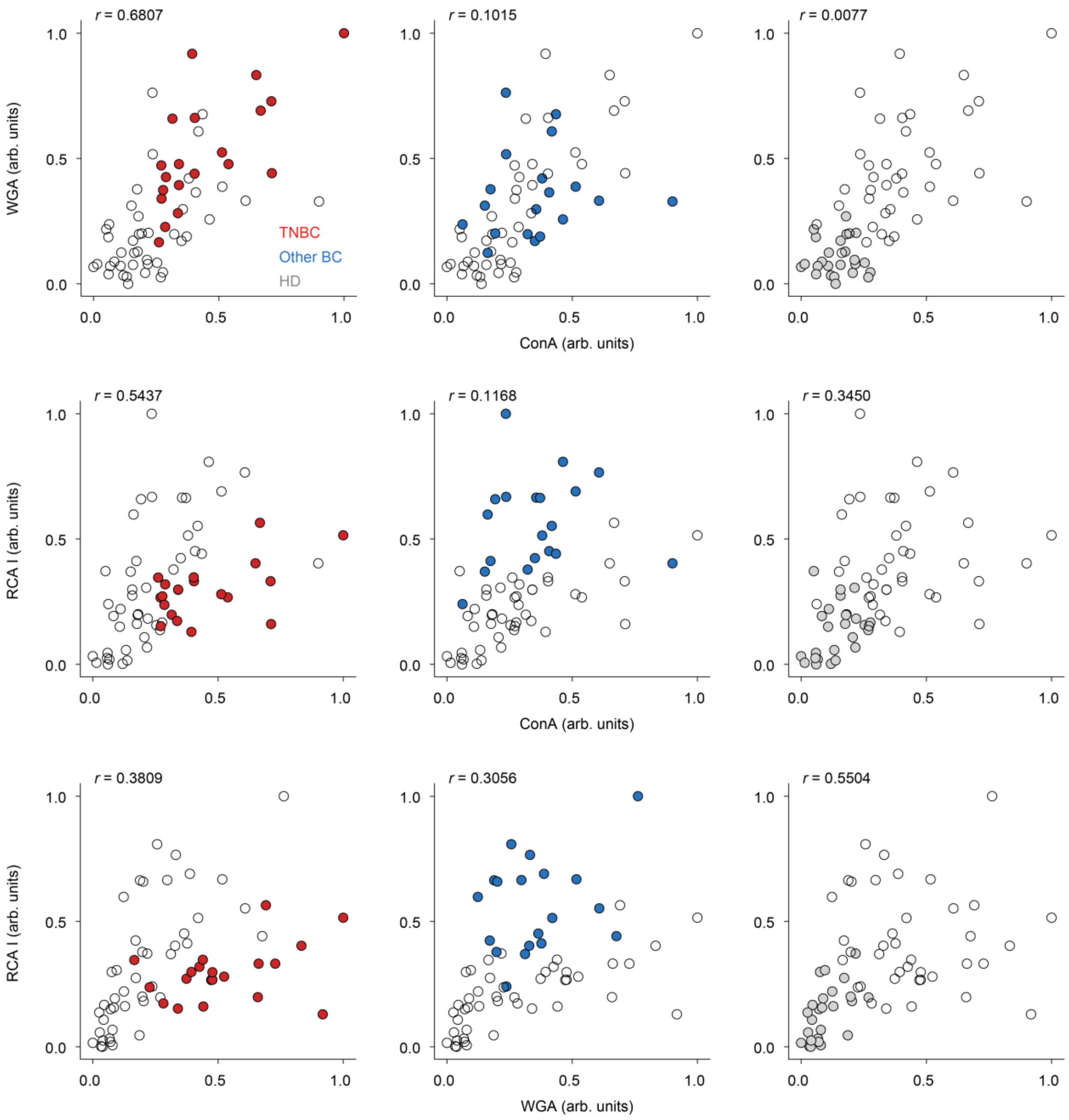

Supplementary Fig. 26 | Correlation of signal intensities between each pair of lectins in TNBC diagnosis within the training cohort. Gray dots represent HD (n = 25), blue dots represent other BC subtype (n = 19), red dots represent TNBC (n = 20). Pearson's r is indicated. Source data are provided as a Source Data file.

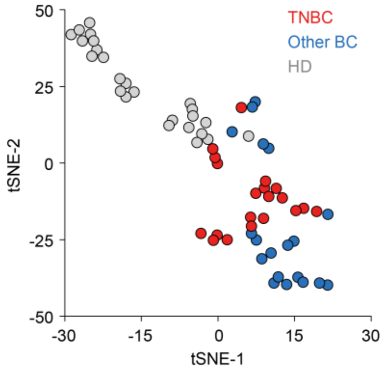

Supplementary Fig. 27 | t-SNE visualization of TNBC, other BC subtypes and HD classification by 3 lectins in the training cohort. Gray dots represent HD (*n* = 25), blue dots represent other BC subtype (*n* = 19), red dots represent TNBC (*n* = 20). Source data are provided as a Source Data file.

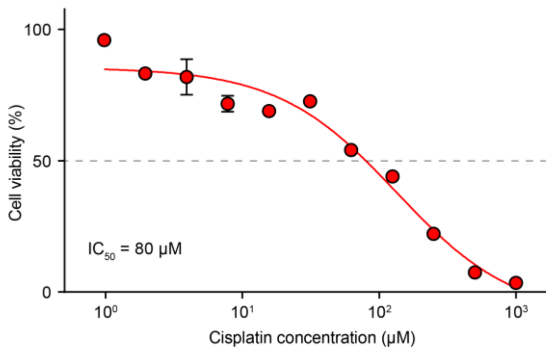

Supplementary Fig. 28 | Cell viability and IC50 value for cisplatin treatment of MDA-MB-231 cells (*n* = 3 samples for each cisplatin concentration). IC50 value (half-maximal inhibitory concentration) is indicated. Error bars represent the mean ± s.d. Source data are provided as a Source Data file.

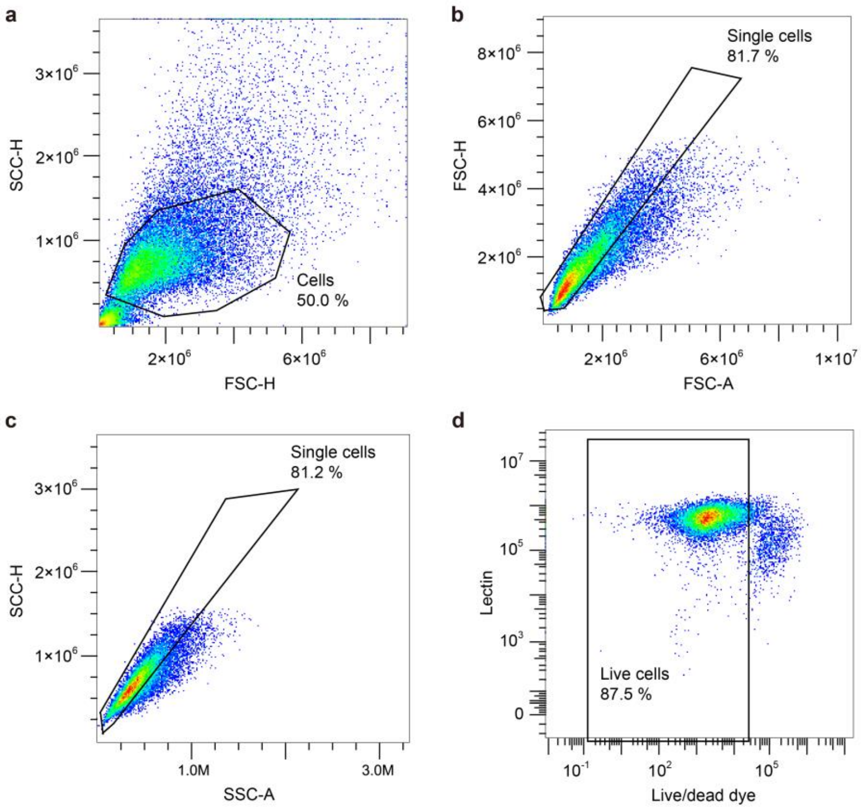

Supplementary Fig. 29 | Gating strategy for flow cytometry analysis of lectin-labeled MDA-MB-231 cells after cisplatin treatment. a, Cells gated based on FSC-H vs SSC-H. **b-c**, Single cells gated based on FSC-A vs FSC-H (**b**) and SSC-A vs SSC-H (**c**). **d**, Live cells further gated on the less stained population after labelling with live/dead dye. Cell population abundances are indicated.

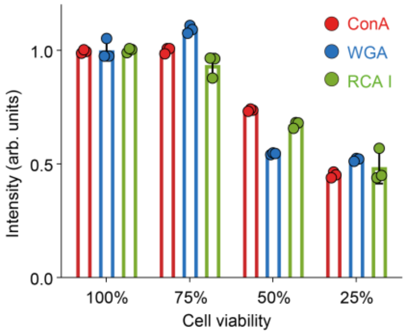

Supplementary Fig. 30 | Flow cytometry analysis of lectin-labeled MDA-MB-231 cells after cisplatin treatment. Cell viabilities of 100 % (untreated), 75 %, 50 % and 25 % were selected. The intensity was normalized against the untreated samples (n = 3 samples for each cell viability). Error bars represent the mean ± s.d. Source data are provided as a Source Data file.

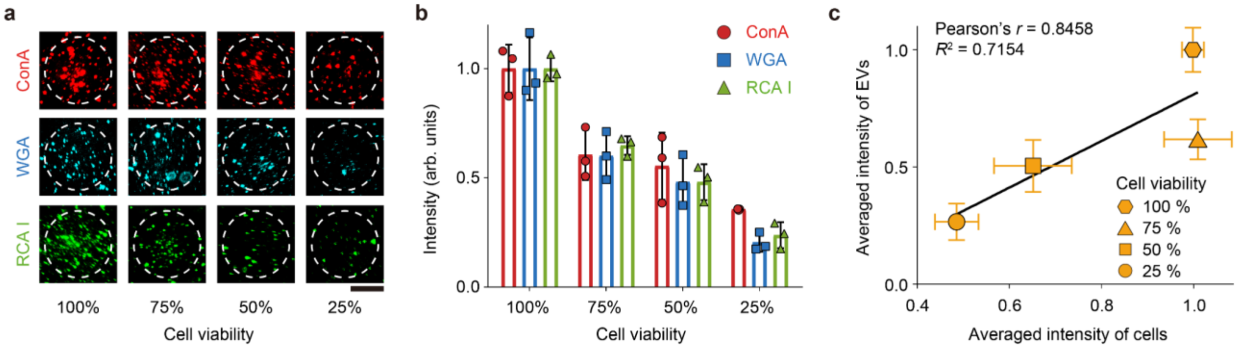

Supplementary Fig. 31 | EVLET analysis of MDA-MB-231 EVs after cisplatin treatment. a, Fluorescence images of the accumulated EVs conjugated with different lectins at various cell viabilities of 100 % (untreated), 75 %, 50 % and 25 %. Scale bar, 50 µm. **b,** Fluorescence intensity of MDA-MB-231 EVs showing the viability-dependent decline in EV surface glycans. The intensity was normalized against the untreated samples (*n* = 3 samples for each cell viability). **c,** Correlation between the arithmetic average of the signal intensities of 3 lectins for EVs and parental cells (*n* = 3 samples for each cell viability). Pearson's *r* and R square (R^2) are indicated. Pearson's correlation was determined by two-sided test (**c**).Error bars represent the mean ± s.d in (**b**, **c**). Source data are provided as a Source Data file.

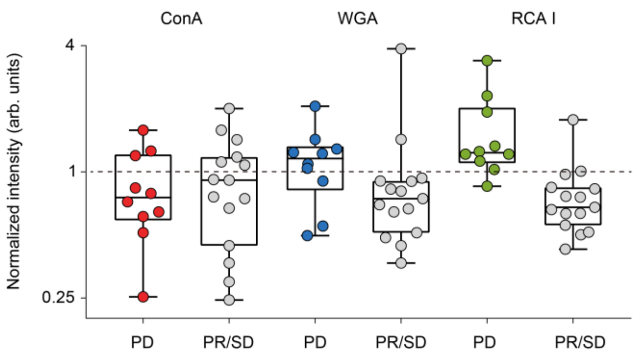

Supplementary Fig. 32 | Individual lectins for differentiating PD (*n* = 10) and PR/SD (*n* = 15) groups. The signal intensity by lectins was normalized by the baseline value for each individual patient for convenient comparison across different patients. The central line, box and error bar indicate the median, inter-quartile range (Q1 and Q3) and min-max range, respectively. Source data are provided as a Source Data file.

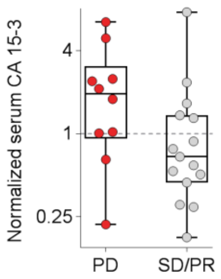

Supplementary Fig. 33 | Serum CA 15-3 level for differentiating PD (*n* = 10) and PR/SD (*n* = 15) groups. The serum CA 15-3 level was normalized by the baseline value for each individual patient for convenient comparison across different patients. The central line, box and error bar indicate the median, inter-quartile range (Q1 and Q3) and min-max range, respectively. Source data are provided as a Source Data file.

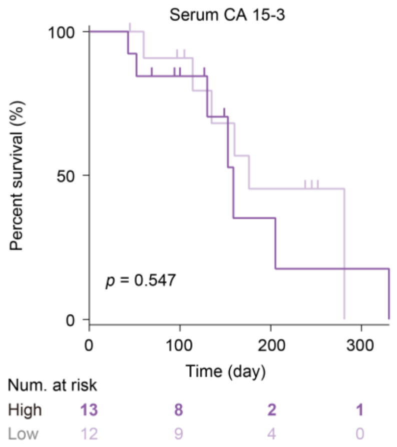

Supplementary Fig. 34 | Serum CA 15-3 for prediction of PFS in TNBC. The significance of difference was calculated by a two-sided log-rank test. *P* value is indicated. Source data are provided as a Source Data file.

Supplementary Table 1 | Summary of glycan specificities recognized by lectins.

Lectin abbreviation	Name	Glycan specificity	Size
**ConA**	Concanavalin A	αMan, αGlc	104 KDa
**WGA**	Wheat germ agglutinin	GlcNAc, SA	36 KDa
**RCA I**	Ricinus communis agglutinin 1	Gal	120 KDa
**SBA**	Soybean agglutinin	α/βGalNAc	110 KDa
**UEA I**	Ulex europaeus agglutinin 1	αFuc	63 KDa

Glycan abbreviations:
Glc: glucose; GlcNAc: N-Acetylglucosamine;
Gal: galactose; GalNAc: N-Acetylgalactosamine;
Man: mannose; SA: sialic acid; Fuc: fucose.

Supplementary Table 2 | Size distribution of EVs.

EV source	Isolation method	Size measurement	Size distribution
**MDA-MB-231 cell**	UC	Cryo-EM	95 % EVs > 50 nm, mode size of 120 nm[S1]
**B16-F1 cell**	UC	Cryo-EM	Almost all EVs > 50 nm, mode size of 100 nm[S2]
**Jurkat, THP-1, U937, MiaPaCa cell lines**	UC	TRPS	All EVs: > 60 nm, mode sizes: ~ 100 nm[S3]
**BT-474 cell**	UC	TRPS	All EVs > 50 nm, mode size of 60 – 70 nm[S4]
**Human plasma**	UC	Cryo-EM	Over 80 % EVs > 50 nm[S5]
**Human plasma**	NA	Cryo-EM	Over 80 % EVs > 100 nm, mode size: 100 – 200 nm[S6]
**Human plasma**	UC	TRPS	Almost all EVs > 50 nm, mode size ~ 100 nm[S7]
**Human plasma**	SEC	TRPS	mode size: 55 – 73 nm[S8]

UC: ultracentrifugation

SEC: size exclusion chromatography

TRPS: tunable resistive pulse sensing

Supplementary Table 3 | Comparison between ultracentrifugation (UC), size exclusion chromatography (SEC), tangential flow filtration (TFF) and vibrating membrane filtration (VMF) in processing plasma/serum samples.

	UC	SEC	TFF	VMF (present study)
**Recovery rate**	*1 % (present)*<br>< 5 %[S9, 10]	*39.3 % (present)*<br>60 % – 85 %[S11, 12]	60 % – 80 %[S13, 14, 15]	*22.1 % (present)*
**Purity (particles/μg protein)**	*1.8×10^7 (present)*<br>10^7 – 2×10^8 [S13, 16, 17]	*1.3×10^9 (present)*<br>0.5×10^9 – 6×10^9 [S17, 18, 19]	10^7 – 1.2×10^8 [S13, 15]	*9.3×10^8 (present)*
**Removal rate of contaminants**	*Total protein: 99.3 %*<br>*LDL: 96.7 %*<br>*VLDL: 98.6 %*<br>*(present)*	*Total protein: 99.4 %*<br>*LDL: 95.7 %*<br>*VLDL: 88.7 %*<br>*(present)*	Total protein: 98.4 %[S20]	*Total protein: 99.5 %*<br>*LDL: > 99.1 %*<br>*VLDL: >98.9 %*<br>*(present)*
**Sample volume**	*2 mL (present)*<br>1 mL – 4 mL[S16, 17]	*2 mL (present)*<br>0.15 mL – 4 mL[S16, 18, 21, 22, 23]	0.03 – 0.6 mL[S13, 14, 15]	*0.002 mL (present)*
**Processing time**	*3 h (present)*<br>1.4 – 3.5 h[S16, 18, 24, 25]	*3.5 h (present)*<br>1.5 – 4 h[S24, 26]	< 0.7 – 3 h[S13, 14, 15]	*0.16 h (present)*

Supplementary Table 4 | Summary of training cohort for TNBC diagnosis.

Characteristic	TNBC	Other BC subtypes	HD	Total
**Total cases**	20	19	25	64
**Subtypes**				
**HR+ HER2-**	–	12	–	12
**HR+ HER2+**	–	3	–	3
**HR- HER2+**	–	4	–	4
**TNBC**	20	–	–	20
**Age (year)**				
**Median (range)**	52 (35 –80)	52 (35 – 70)	45 (23 – 64)	50 (23 – 80)
**Stage**				
**I**	1	2	–	3
**II**	4	3	–	7
**III**	2	1	–	3
**IV**	8	12	–	20
**Unknown**	5	1	–	6
**Serum CA 15-3 (U mL^{-1})**				
**Median (range)**	14.8 (4.6 – 208.0)	33.2 (6.4 – 3000)	–	20.3 (4.6 – 3000)

Supplementary Table 5 | Summary of validation cohort for TNBC diagnosis.

Characteristic	TNBC	Other BC subtypes	HD	Total
**Total cases**	8	11	13	32
**Subtypes**				
**HR+ HER2-**	–	5	–	5
**HR+ HER2+**	–	3	–	3
**HR- HER2+**	–	3	–	3
**TNBC**	8	–	–	8
**Age (year)**				
**Median (range)**	54 (28-70)	53 (39-68)	54 (43 – 65)	54 (28-70)
**Stage**				
**I**	0	1	–	1
**II**	2	0	–	2
**III**	0	1	–	1
**IV**	4	9	–	13
**Unknown**	2	0	–	2
**Serum CA 15-3 (U mL^{-1})**				
**Median (range)**	8.0 (2.8 –131.0)	15.1 (6.2 – 164.2)	–	7.3 (2.8 – 164.2)

Supplementary Table 6 | Summary of TNBC monitoring cohort. The patients received chemotherapy treatment in accordance with Standard of Care.

Characteristic	Responder	Non-responder	Total
**Total cases**	5	8	13
**Age (year)**			
**Median (range)**	43 (28-60)	51 (31-80)	47 (28-80)
**Type of therapy**			
**Platinum number (%)**	3 (60%)	2 (25%)	5 (38.5%)
**Taxane number (%)**	2 (40%)	3 (37.5%)	5 (38.5%)
**Navelbine number (%)**	3 (60%)	3 (37.5%)	6 (46.2%)
**Capecitabine number (%)**	0 (0%)	2 (25%)	2 (15.4%)
**Number of rounds**			
**Median (range)**	4 (2-12)	4 (2-8)	4 (2-12)

Supplementary Table 7 | Summary of TNBC monitoring cohort in terms of different response groups.

Characteristic	PD	SD	PR	Total
**Total cases**	10	5	10	25
**Age (year)**				
**Median (range)**	49 (31 – 80)	51 (28 – 80)	45 (28 – 80)	47 (28 – 80)
**Serum CA 15-3 (U mL^{-1})**				
**Median (range)**	22.9 (6.7 – 116)	26.2 (8.9 – 137)	14.1 (5.8 – 42.4)	22.7 (5.8 – 137)

Supplementary Table 8 | Summary of TNBC prognosis cohort. The patients received chemotherapy treatment in accordance with Standard of Care.

Characteristic	PD	Censored	Total
**Total cases**	13	12	25
**Age (year)**			
**Median (range)**	51 (37 – 80)	47 (28 – 67)	50 (28 – 80)
**Serum CA 15-3 (U mL^{-1})**			
**Median (range)**	16.4 (2.8 – 163.0)	10.5 (5.5 – 61.0)	12.6 (2.8 – 163.0)
**Type of therapy**			
**Platinum number (%)**	7 (58.3%)	6 (50%)	13 (52%)
**Taxane number (%)**	5 (38.5%)	7 (58.3%)	12 (48%)
**Navelbine number (%)**	4 (30.8%)	3 (25%)	7 (28%)
**Capecitabine number (%)**	4 (30.8%)	2 (16.7%)	6 (24%)
**Gemcitabine number (%)**	1 (7.7%)	1 (8.3%)	2 (8%)
**Number of rounds**			
**Median (range)**	6 (2 – 10)	4 (2 – 12)	6 (2 – 12)

Supplementary Table 9 | Hazard ratio by univariate Cox proportional hazard regression. Two-sided statistical test was used without adjustments for multiple comparisons.

Variable	Hazard ratio	*P* value	95 % CI
**TNBC^{EGP} signature**	6.17	0.021	1.32 – 28.87
**Age**	1.03	0.235	0.98 – 1.07
**Serum CA 15-3**	1.02	0.032	1.01 – 1.04
**Ki67**	0.99	0.559	0.96 – 1.02

References

S1. Rontogianni S, *et al.* Proteomic profiling of extracellular vesicles allows for human breast cancer subtyping. *Commun Biol* **2**, 325 (2019).

S2. Muhsin-Sharafaldine M-R, Saunderson SC, Dunn AC, Faed JM, Kleffmann T, McLellan AD. Procoagulant and immunogenic properties of melanoma exosomes, microvesicles and apoptotic vesicles. *Oncotarget* **7**, 56279–56294 (2016).

S3. Osteikoetxea X, *et al.* Differential detergent sensitivity of extracellular vesicle subpopulations. *Org Biomol Chem* **13**, 9775-9782 (2015).

S4. Lane RE, Korbie D, Anderson W, Vaidyanathan R, Trau M. Analysis of exosome purification methods using a model liposome system and tunable-resistive pulse sensing. *Sci Rep* **5**, 7639 (2015).

S5. Tutanov O, Proskura K, Kamyshinsky R, Shtam T, Tsentalovich Y, Tamkovich S. Proteomic Profiling of Plasma and Total Blood Exosomes in Breast Cancer: A Potential Role in Tumor Progression, Diagnosis, and Prognosis. *Front Oncol* **10**, 580891 (2020).

S6. Arraud N, *et al.* Extracellular vesicles from blood plasma: determination of their morphology, size, phenotype and concentration. *J Thromb Haemost* **12**, 614-627 (2014).

S7. Pasetto L, *et al.* Decoding distinctive features of plasma extracellular vesicles in amyotrophic lateral sclerosis. *Mol Neurodegener* **16**, 52 (2021).

S8. Diehl JN, *et al.* A standardized method for plasma extracellular vesicle isolation and size distribution analysis. *PLoS One* **18**, e0284875 (2023).

S9. Momen-Heravi F, *et al.* Impact of biofluid viscosity on size and sedimentation efficiency of the isolated microvesicles. *Front Physiol* **3**, 162 (2012).

S10. Baranyai T, *et al.* Isolation of Exosomes from Blood Plasma: Qualitative and Quantitative Comparison of Ultracentrifugation and Size Exclusion Chromatography Methods. *PLoS One* **10**, e0145686 (2015).

S11. Vogel R, *et al.* A standardized method to determine the concentration of extracellular vesicles using tunable resistive pulse sensing. *J Extracell Vesicles* **5**, 31242 (2016).

S12. Lobb RJ, *et al.* Optimized exosome isolation protocol for cell culture supernatant and human plasma. *J Extracell Vesicles* **4**, 27031 (2015).

S13. Han Z, *et al.* Highly efficient exosome purification from human plasma by tangential flow filtration based microfluidic chip. *Sens Actuators B: Chem* **333**, 129563 (2021).

S14. Hua X, *et al.* A double tangential flow filtration-based microfluidic device for highly efficient separation and enrichment of exosomes. *Anal Chim Acta* **1258**, 341160 (2023).

S15. Sunkara V, *et al.* Fully Automated, Label-Free Isolation of Extracellular Vesicles from Whole Blood for Cancer Diagnosis and Monitoring. *Theranostics* **9**, 1851-1863 (2019).

S16. Takov K, Yellon DM, Davidson SM. Comparison of small extracellular vesicles isolated from plasma by ultracentrifugation or size-exclusion chromatography: yield, purity and functional potential. *J Extracell Vesicles* **8**, 1560809 (2019).

S17. Wei R, *et al.* Combination of Size-Exclusion Chromatography and Ultracentrifugation Improves the Proteomic Profiling of Plasma-Derived Small Extracellular Vesicles. *Biol Proced Online* **22**, 12 (2020).

S18. Pang B, *et al.* Quality assessment and comparison of plasma-derived extracellular vesicles separated by three commercial kits for prostate cancer diagnosis. *Int J Nanomedicine* **15**, 10241-10256 (2020).

S19. Stranska R, *et al.* Comparison of membrane affinity-based method with size-exclusion chromatography for isolation of exosome-like vesicles from human plasma. *J Transl Med* **16**, 1 (2018).
S20. Dong L, *et al.* Comprehensive evaluation of methods for small extracellular vesicles separation from human plasma, urine and cell culture medium. *J Extracell Vesicles* **10**, e12044 (2020).
S21. Pesce E, *et al.* Exosomes Recovered From the Plasma of COVID-19 Patients Expose SARS-CoV-2 Spike-Derived Fragments and Contribute to the Adaptive Immune Response. *Front Immunol* **12**, 785941 (2022).
S22. Buschmann D, *et al.* Evaluation of serum extracellular vesicle isolation methods for profiling miRNAs by next-generation sequencing. *J Extracell Vesicles* **7**, 1481321 (2018).
S23. Sidhom K, Obi PO, Saleem A. A Review of Exosomal Isolation Methods: Is Size Exclusion Chromatography the Best Option? *Int J Mol Sci* **21**, 6466 (2020).
S24. Chen Y, *et al.* Exosome detection via the ultrafast-isolation system: EXODUS. *Nat Methods* **18**, 212-218 (2021).
S25. Visan KS, *et al.* Comparative analysis of tangential flow filtration and ultracentrifugation, both combined with subsequent size exclusion chromatography, for the isolation of small extracellular vesicles. *J Extracell Vesicles* **11**, 12266 (2022).
S26. Veerman RE, *et al.* Molecular evaluation of five different isolation methods for extracellular vesicles reveals different clinical applicability and subcellular origin. *J Extracell Vesicles* **10**, e12128 (2021).
