# Supplementary material for: Thermophoretic glycan profiling of extracellular vesicles for triple-negative breast cancer management
Source: Nat Commun. 2024 Mar 14;15:2292. doi: 10.1038/s41467-024-46557-5 (PMC10937950; doi:10.1038/s41467-024-46557-5)
Supplement: Supplementary file 3 — Reporting Summary [file 41467_2024_46557_MOESM3_ESM.pdf]

nature portfolio

Corresponding author(s): Jiashu Sun, Liqin Zhang, Yuguang Wang, Chao Liu

Last updated by author(s): Feb 13, 2024

Reporting Summary

Nature Portfolio wishes to improve the reproducibility of the work that we publish. This form provides structure for consistency and transparency in reporting. For further information on Nature Portfolio policies, see our Editorial Policies and the Editorial Policy Checklist.

Statistics

For all statistical analyses, confirm that the following items are present in the figure legend, table legend, main text, or Methods section.

n/a	Confirmed	
☐	☒	The exact sample size (*n*) for each experimental group/condition, given as a discrete number and unit of measurement
☐	☒	A statement on whether measurements were taken from distinct samples or whether the same sample was measured repeatedly
☐	☒	The statistical test(s) used AND whether they are one- or two-sided<br>*Only common tests should be described solely by name; describe more complex techniques in the Methods section.*
☒	☐	A description of all covariates tested
☒	☐	A description of any assumptions or corrections, such as tests of normality and adjustment for multiple comparisons
☐	☒	A full description of the statistical parameters including central tendency (e.g. means) or other basic estimates (e.g. regression coefficient) AND variation (e.g. standard deviation) or associated estimates of uncertainty (e.g. confidence intervals)
☐	☒	For null hypothesis testing, the test statistic (e.g. *F*, *t*, *r*) with confidence intervals, effect sizes, degrees of freedom and *P* value noted<br>*Give P values as exact values whenever suitable.*
☒	☐	For Bayesian analysis, information on the choice of priors and Markov chain Monte Carlo settings
☒	☐	For hierarchical and complex designs, identification of the appropriate level for tests and full reporting of outcomes
☐	☒	Estimates of effect sizes (e.g. Cohen's *d*, Pearson's *r*), indicating how they were calculated

Our web collection on statistics for biologists contains articles on many of the points above.

Software and code

Policy information about availability of computer code

Data collection	The NTA data were captured using the NTA 3.4 Analytical Software Suite. Fluorescence images were recorded using Micro-Manager 1.4. The TEM data were captured using DigitalMicrograph 3.1. The SEM data were captured using the S-4800 Software. The flow cytometry data were captured using NovoExpress Software.
Data analysis	The TEM data and the SEM data were analyzed using ImageJ 1.53a. Particle spatial distributions were analyzed using Comsol 4.4. The flow cytometry data were analyzed using FlowJo V10. Significance analyses, Pearson correlation, ROC curve construction and AUC calculation were performed using GraphPad Prism 9.5. Kaplan-Meier analysis, log-rank test, LDA (based on package MASS, version 7.3) and Cox regression were performed using R software (version 4.2.3).

For manuscripts utilizing custom algorithms or software that are central to the research but not yet described in published literature, software must be made available to editors and reviewers. We strongly encourage code deposition in a community repository (e.g. GitHub). See the Nature Portfolio guidelines for submitting code & software for further information.

Data

Policy information about availability of data

All manuscripts must include a data availability statement. This statement should provide the following information, where applicable:

- Accession codes, unique identifiers, or web links for publicly available datasets
- A description of any restrictions on data availability
- For clinical datasets or third party data, please ensure that the statement adheres to our policy

All data generated in this study are provided in the paper, Supplementary Information and Source Data file. The expression data of mRNA transcripts in the TCGA-BRCA cohort used in this study are available in the Cancer Genome Atlas (TCGA) database under accession code phs000178 (https://portal.gdc.cancer.gov). Source data are provided with this paper.

Research involving human participants, their data, or biological material

Policy information about studies with human participants or human data. See also policy information about sex, gender (identity/presentation), and sexual orientation and race, ethnicity and racism.

Reporting on sex and gender	Our study exclusively involves female patients due to its focus on breast cancer.
Reporting on race, ethnicity, or other socially relevant groupings	Our study only includes Chinese patients.
Population characteristics	All the participants were female. For detection of TNBC, other BC subtypes and healthy donors (HDs), 96 participants were older than 23 years and younger than 80 years, including 28 patients with TNBC, 30 patients with other BC subtypes, and 38 age-matched HDs. Prior to sample collection, the BC patients received no salvage treatment. Relevant information on the human participants in the TNBC detection cohort are presented in Supplementary Tables 4-5. For treatment monitoring, 25 samples were from 13 TNBC patients that were older than 28 years and younger than 80 years after 2 to 12 rounds of treatment. Relevant information on the human participants in the monitoring cohort are presented in Supplementary Tables 6-7. For PFS prediction, 25 TNBC patients were older than 28 years and younger than 80 years. Relevant information on the human participants in the prognosis cohort are presented in Supplementary Table 8.
Recruitment	BC patients and healthy donors were recruited from the Fifth Medical Centre, Chinese PLA General Hospital. Inclusion criteria for the recruitment were as follows: females aged 18 – 80 years; patients diagnosed with breast cancer through immunohistochemistry or FISH testing (including triple-negative, luminal A, luminal B, and HER2 overexpression types of breast cancer), or healthy subjects determined through blood biochemical and urine tests; no severe underlying diseases; willing to participate. The exclusion criteria included: individuals with severe other diseases; pregnant or breastfeeding women. The study complied with all relevant ethical regulations and was approved by the Ethics Committee of the Fifth Medical Center of PLA General Hospital. All individuals were anonymized, and only gender, age, pathological diagnosis, treatment plan and treatment response were recorded. No self-selection criteria bias for patient populations was anticipated.
Ethics oversight	The study is conducted in accordance with Declaration of Helsinki protocol and in accordance with the terms and conditions of the ethical approval from the Ethics Committee of the Fifth Medical Center of PLA General Hospital. All participants provided written informed consent.

Note that full information on the approval of the study protocol must also be provided in the manuscript.

Field-specific reporting

Please select the one below that is the best fit for your research. If you are not sure, read the appropriate sections before making your selection.

☒ Life sciences ☐ Behavioural & social sciences ☐ Ecological, evolutionary & environmental sciences

For a reference copy of the document with all sections, see nature.com/documents/nr-reporting-summary-flat.pdf

Life sciences study design

All studies must disclose on these points even when the disclosure is negative.

Sample size	For TNBC detection, n = 96 clinical samples were used to differentiate TNBC from other BC subtypes and HDs. For treatment monitoring, n = 38 samples from 25 TNBC patients were used to differentiate PD from PR/SD or differentiate responders from non-responders. The sample sizes were determined by sample availability. These sample sizes were sufficiently large for performing LDA classification based on previous experience that sample size is not less than 10 times the features (i.e., the fluorescence intensity of 3 lectins in EVLET).
Data exclusions	No data were excluded.
Replication	The successful replication for EVLET measurement in randomly selected BC and HD samples was shown by low inter-batch variations of less than 15 % based on 3 independent experiments for each sample (see Supplementary Fig. 21). Having proved the reproducibility, EVLET measurement of the remained clinical samples was performed without replication. For optimization in operation conditions of EVLET, 3

independent experiments were conducted for each applicable condition. For evaluation and comparison in EVLET performance across different sample groups, 3 independent experiments were conducted using 3 different batches of samples for each sample group. All attempts at replication were successful.

Randomization

For detection of TNBC, other BC subtypes and HD, samples were randomly assigned to the training or validation cohort. For treatment monitoring and prognosis, all samples were not divided given the sample availability and thus randomization was not involved. For experiments other than the clinical cohort study, the randomization was not relevant because samples were intentionally prepared and assigned to different experimental groups for the aim of EVLET optimization or performance evaluation.

Blinding

The investigators who performed EVLET assay were blinded to cell line type, patient selection, and the diagnostic results and treatment responses of all cohorts. No blinding was used for performance comparison between EVLET and other methods because the same samples were used.

Reporting for specific materials, systems and methods

We require information from authors about some types of materials, experimental systems and methods used in many studies. Here, indicate whether each material, system or method listed is relevant to your study. If you are not sure if a list item applies to your research, read the appropriate section before selecting a response.

Materials & experimental systems

n/a	Involved in the study	
☐	☒	Antibodies
☐	☒	Eukaryotic cell lines
☒	☐	Palaeontology and archaeology
☒	☐	Animals and other organisms
☒	☐	Clinical data
☒	☐	Dual use research of concern
☒	☐	Plants

Methods

n/a	Involved in the study	
☒	☐	ChIP-seq
☐	☒	Flow cytometry
☒	☐	MRI-based neuroimaging

Antibodies

Antibodies used

anti-CD81 antibody (Supplier name: Abcam, Catalog number: #ab109201)
anti-Apolipoprotein B (ApoB) antibody (Supplier name: Abcam, Catalog number: #ab139401)
anti-rabbit IgG (Supplier name: Abcam, Catalog number: #ab6721)
anti-HSP90 antibody (Supplier name: Sino Biological, Catalog number: 207258-T32)
anti-calnexin antibody (Supplier name: Bioss, Catalog number: bsm-52639R)

Validation

All antibodies used are commercially available and were validated either by manufacture or used in published research papers. For example:
Leidal A M, Huang H H, Marsh T, et al. The LC3-conjugation machinery specifies the loading of RNA-binding proteins into extracellular vesicles[J]. Nature cell biology, 2020, 22(2): 187-199.
Zhang C, Huo X, Zhu Y, et al. Electrodeposited magnetic nanoporous membrane for high-yield and high-throughput immunocapture of extracellular vesicles and lipoproteins[J]. Communications Biology, 2022, 5(1): 1358.
Słabicki M, Kozicka Z, Petzold G, et al. The CDK inhibitor CR8 acts as a molecular glue degrader that depletes cyclin K[J]. Nature, 2020, 585(7824): 293-297.
Wang X, Xing C, Li G, et al. The key role of proteostasis at mitochondria-associated endoplasmic reticulum membrane in vanadium-induced nephrotoxicity using a proteomic strategy[J]. Science of The Total Environment, 2023, 869: 161741.

Eukaryotic cell lines

Policy information about cell lines and Sex and Gender in Research

Cell line source(s)

Human breast cancer cell lines (MDA-MB-231, MDA-MB-453, BT-474, and MCF-7) and human benign breast epithelial cell line MCF-10A were obtained from ATCC.

Authentication

Cell types were authenticated via STR profiling and matched their publicly available STR profiles. All cell lines were used without any modification.

Mycoplasma contamination

All cell lines were negative for mycoplasma contamination.

Commonly misidentified lines
(See ICLAC register)

No commonly misidentified cell lines were used.

Plants

Seed stocks: n/a

Novel plant genotypes: n/a

Authentication: n/a

Flow Cytometry

Plots

Confirm that:

- [x] The axis labels state the marker and fluorochrome used (e.g. CD4-FITC).
- [x] The axis scales are clearly visible. Include numbers along axes only for bottom left plot of group (a 'group' is an analysis of identical markers).
- [x] All plots are contour plots with outliers or pseudocolor plots.
- [x] A numerical value for number of cells or percentage (with statistics) is provided.

Methodology

Sample preparation: Cells were collected and washed twice with 1×PBS. The cells were resuspended in PBS and incubated with FITC-conjugated lectins for 1 h at 4 °C, followed by labelling with live/dead dye (Fixable Viability Dye eFluor™ 660, 1/1000) for 30 min at 4 °C. The labeled cells were washed by 1× PBS (centrifugation at 500×g for 5 min) twice and suspended in 800 µL 1× PBS containing 2 % FBS prior to the flow cytometric characterization.

Instrument: ACEA NovoCyte, Agilent Technologies

Software: FlowJo (V10), NovoExpress

Cell population abundance: The abundance was 12%-60% after gating.

Gating strategy: Single cells gated based on FSC-A vs FSC-H and SSC-A vs SSC-H. Live cells further gated on the less stained population after labelling with live/dead dye.

- [x] Tick this box to confirm that a figure exemplifying the gating strategy is provided in the Supplementary Information.
